# Impact of a well-developed primary care system on the length of stay in emergency departments in the Netherlands: a multicenter study

**DOI:** 10.1186/s12913-016-1400-z

**Published:** 2016-04-26

**Authors:** Wendy A. M. H. Thijssen, Nicole Kraaijvanger, Dennis G. Barten, Marleen L. M. Boerma, Paul Giesen, Michel Wensing

**Affiliations:** Emergency Department, Catharina Hospital, Michelangelolaan 2, Postbus 1350, 5602ZA Eindhoven, The Netherlands; Radboud University Medical Centre, Scientific Center for Quality of Healthcare, Nijmegen, The Netherlands; Emergency Department, Rijnstate Hospital, Arnhem, The Netherlands; Emergency Department, VieCuri Medical Center, Venlo, The Netherlands; Emergency Department, Elisabeth Hospital, Tilburg, The Netherlands

**Keywords:** Crowding, Primary care physician, Emergency department, Length of stay

## Abstract

**Background:**

The Netherlands has a well-developed primary care system, which increasingly collaborates with hospital emergency departments (EDs). In this setting, insight into crowding in EDs is limited. This study explored links between patients’ ED Length of Stay (LOS) and their care pathways.

**Methods:**

Observational multicenter study of 7000 ED patient records from 1 February 2013. Seven EDs spread over the Netherlands, representing overall Dutch EDs, were included. This included three EDs with and four EDs without an integrated primary-care-physician (PCP) cooperative, forming one Emergency Care Access Point (ECAP). The main outcome was LOS of patients comparing different care pathways (origin and destination of ED attenders).

**Results:**

The median LOS of ED attenders was 130.0 min (IQR 79.0–140.0), which increased with patients’ age. Random coefficient regression analysis showed that LOS for patients referred by medical professionals was 32.9 min longer compared to self-referred patients (95 % CI 27.7–38.2 min). LOS for patients admitted to hospital was 41.2 min longer compared to patients followed-up at the outpatient clinic (95 % CI 35.3–46.6 min), 49.9 min longer compared to patients followed-up at the PCP (95 % CI 41.5–58.3 min) and 44.6 min longer compared to patients who did not receive follow-up (95 % CI 38.3–51.0 min). There was no difference in LOS between hospitals with or without an ECAP.

**Conclusions:**

With 130 min, the median LOS in Dutch EDs is relatively short, comparing to other Western countries, which ranges from 176 to 480 min. Although integration of EDs with out-of-hours primary care was not related to LOS, the strong primary care system probably contributed to the overall short LOS of ED patients in the Netherlands.

## Background

Crowding of Emergency Departments (EDs) is a growing concern in many countries, leading to increasing lengths of stay (LOS) in the ED. Long LOS has been associated with decreased patient satisfaction, treatment delays, patients leaving without being seen and ambulance diversions. Non-urgent visits, influenza season and hospital bed shortages are some of the factors that have been identified as causes for crowding, [1–5]. Because non-urgent ED visits are also associated with ED crowding and policies to redirect these patients to primary care might contribute to a reduction of LOS [6, 7]. The success of redirecting patients is influenced by the structure of the national healthcare system and the position of primary healthcare. Worldwide, different models of organized healthcare systems are used to redirect patients to primary care services, each having its unique effect on the ED patient population [8–11].

In the Netherlands, primary healthcare is well-developed and accessible for patients 24 h a day. During office-hours patients can present at their own primary care physician (PCP) practice, usually on the same day. After-hours, primary care practitioners provide emergency services through large scale PCP Cooperatives [12]. There is an increasing trend towards implementing Emergency Care Access Point’s (ECAP); a place where EDs and PCPs work together, creating one desk where triage decides if the patient will be seen by a PCP or in the ED [13]. The main goal is redirecting the non-urgent self-referrals to the PCP and having the PCP function as a gatekeeper for emergency departments visits. The implementation of the ECAP has led to a decrease of self-referred ED patients and changed the acuity and admission rates of presenting ED patients [14–18]. Despite growing concerns of increasing LOS in the Netherlands, there was a shortage of data on LOS at EDs and associated factors. In particular, there was no research available that looked at patients’ care pathways, that is origin and destination of patients attending the ED.

This study aimed to provide insight in the LOS in EDs and to explore links with patients’ care pathways in the Netherlands, a country with a well-developed primary health care system.

## Methods

### Study design and setting

This was an observational multicenter study of 7000 patient records of EDs in the Netherlands. To make sure our data represented Dutch EDs, patients were sampled from seven EDs spread over the Netherlands, including small urban EDs, large inner city EDs and EDs with and without an ECAP. The patient samples comprised the first 1000 attending patients from February 1st 2013 onwards. Patients who were registered in the ED system, but received healthcare at the PCP cooperative, an outpatient clinic or directly went to the obstetric ward or the cardiac emergency department, were excluded. The average time to include 1000 patients per hospital was 12.8 days (9–17 days). Since there are seasonal effects on LOS, we choose to collect data in the winter months, where LOS overall is longest. This was to compare how the Dutch LOS would compare to international LOS, i.e. the United States, Canada and the United Kingdom.

### Methods and measurements

All hospital EDs had digital registration systems, and the extracted data were put anonymously and numbered in a database. A standardized format was provided to each hospital to ensure that the provided data was comparable. The participating hospitals provided descriptive information regarding the use of a triage system, total annual ED admission over 2012 and the presence of an ECAP. Furthermore, descriptive data were collected, regarding the number of hospital beds, total annual hospital admissions, mean length of hospital stay and the adherence area when available.

Besides LOS, the measures included: date and time of arrival and departure, sex, age, acuity (triage category), trauma or non-trauma related, origin (self-referred, referred by PCP, ambulance, via the radiology department, other) and destination (admitted to hospital, out-patient clinical follow-up, PCP follow-up, no follow-up and other).

Patients who were referred by a PCP and arrived by ambulance comprised a separate category registered in the digital systems. Depending on individual hospital systems, they could either be in the PCP group or in the ambulance group. We therefore combined the two groups and classified these patients as referred by medical professionals. During office-hours PCPs in the Netherlands have the option to refer patients directly to the radiology department for a diagnostic work-up (x-ray or ultrasound). Some ECAPs also have this option after-hours. A radiologist reads the obtained images and either refers the patient to the ED when abnormalities are found or back to the PCP. Because a shorter ED LOS was expected for this group, patients that attended the ED via the radiology department were separately categorized.

### Outcomes

The primary outcome was overall patients’ length of stay at the ED. Secondary outcomes were length of stay for different patients origin (self-referred patients, patients referred by medical professionals and patients referred by the radiology department) and patients follow-up (Admission, follow-up at the out patient clinic, follow-up with the PCP or no follow-up).

### Analysis

All data were checked for integrity and entered in a database. When data about the LOS was missing or appeared to be outliers, the contact person of the specific hospital was contacted and the missing data was hand searched, corrected if needed and added to the database. For data analysis we used IBM SPSS Statistics Version 19. Descriptive statistics (totals, medians, 95 % CI, interquartile range) were used to describe LOS, patient characteristics, and care pathways. We explored whether patient’s LOS was related to origin, destination, time of presentation and the presence of an ECAP. Because the ECAPs only operate after-hours we analyzed LOS comparing ECAP and non-ECAP hospitals only in the after-hours period.

Random coefficient regression modeling was used to explore links of LOS with patients’ age, sex and whether or not patients presented with a trauma related problem. These patient-related measures were included as fixed effects. In a separate regression model we explored the links of LOS with time of presentation, origin, follow up and the presence of an ECAP both separately and combined with age, sex and a trauma related problem (all as fixed factors). Hospital was included as a random factor for all analysis except for the ECAP analysis. *P*-value < 0.05 was considered significant.

## Results

### Hospital characteristics

Table 1 provides descriptive information on the seven participating hospitals. These were spread over the country, varied in size, and included the two largest EDs of the Netherlands.

**Table 1:** Hospital and ED characteristics

		Overview hospitals							
		Hospital 1	Hospital 2	Hospital 3	Hospital 4	Hospital 5	Hospital 6	Hospital 7	Total
	Urbanization	Urban	Urban	Inner-city	Urban	Urban	Inner-city	Urban	--
	Geographic region	Middle	South-west	North-West	Middle	South	West	South East	--
	Population served	460,000	500,000	n/a	n/a	250.000	n/a	280.000	--
	Hospital beds^b^	955^c^	545	555	663	696	654	479	479–955 ^c^
	Total admissions^b^	26,784	26,957	26,022	31,563	28,988	38,861	23.553	202.728
	ED admissions of total hospital admissions (%)^b^	46.2	35.9	30.1	28.6	38.0	32.0	38.0	35.2
	Mean length hospital stay (days)^b^	5.4	5.1^a^	5.0	4.5^a^	4.8	4.3	4.9	4.3–5.4
ED	Total patients per year^b^	36.721	28.234	48.978	24.365	32.132	43.362	26.661	240.453
	Total of ED admissions^b^	12.383	9.678	7.822	9.030	11.027	12.449	8.961	71.350
	ED admissions of total ED presentations (%)^b^	33.7	34.3	16.0	37.1	34.3	28.7	33.6	29.7
	EP present 24/7	Yes	Yes	No	Yes	No	No	No	-
	ECAP	No	No	No	Yes	Yes	Yes	No	-
	Triage System	MTS	ESI	ESI	MTS	NTS	MTS+	MTS	-

^a^N/A in year report, calculated (Known admissions and length of hospital stay), ^b^table presenting numbers of the year 2012, ^c^also includes daycare beds

MTS stands for Manchester triage system, ESI for Emergency Severity Index and NTS for Netherlands Triage Standard

Three hospitals were tertiary cardiac referral centers that performed primary cardiac interventions (PCI). One hospital was a level one-trauma center and three hospitals had 24/7 emergency physicians staffed. Together the hospitals treated 240.453 patients in their EDs in 2012, of which 71.350 patients were admitted to the hospital. This equals an admission rate of 29.7 % of all ED attendances and makes up 35.2 % of total hospital admissions. The average length of hospital admissions ranged from 4.3 to 5.4 days.

Three different triage systems were in use: the Manchester Triage System (MTS), Emergency Severity Index (ESI) and the Netherlands Triage Standard (NTS). There were three hospitals with an ECAP and four without an ECAP.

### Patient characteristics

Of the 7000 included patients, 51.9 % was male and the mean age was 47.0 years (median 49.0, SD 25.6). The majority of patients presented during weekdays (41.9 %) compared to evenings (30.0 %), nights (13.1 %) and weekend days (15.0 %). Of all ED attendances, 32.0 % presented with a trauma related problem. Overall, 36.3 % of ED attendances were admitted to the hospital, 28.5 % were followed up in an outpatient clinic, 9.3 % were referred to their PCP for follow-up and 20.7 % did not require any follow-up (Table 2).

**Table 2:** Patient characteristics and care pathways (*n* = 7000)

		Mean of all hospitals	Lowest-highest value per hospital
Male (%)		51.9	48.4–56.2
Age (%)	0–5 years	7.0	1.7–9.5
	6–18 years	9.3	5.6–10.5
	19–30 years	14.8	11.1–20.6
	31–50 years	20.8	17.0–26.0
	51–65 years	19.9	18.0–22.1
	66–85 years	23.1	12.0–26.8
	>85 years	5.1	3.0–8.4
Presentation (%)	Weekdays^a^	41.9	37.3–50.4
	Evenings^b^	30.0	28.5–31.9
	Nights^c^	13.1	12.2–15.0
	Weekend days^a^	15.0	7.1–19.6
Origin (%)* *N* = 6908^d^	Self-referred patients	21.2	9.4–51.2
	Referred by medical professionals^e^	59.9	38.3–77.2
	Referred by the radiology department	3.3	3–4.8
Follow-up (%)*	Hospital Admission	36.3	18.5–43.5
	Out patient Clinic	28.5	15.7–36.4
	Primary Care Physician	9.3	1.9–39.5
	None	20.7	9.0–26.1
Trauma (%)		32.0	25.4–36.0

*Does not add up to 100 % due to other options not shown in the table ^a^8 am-5 pm, ^b^5 pm-12 am, ^c^12 am-8 am, ^d^there were 92 missing data on patients origin

^e^Includes ambulance and PCP referred patients

### Length of stay

**Fig. 1:**
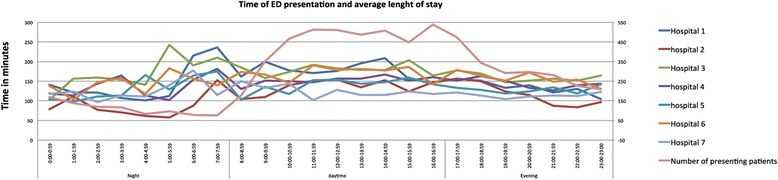
Mean length of stay of all ED patients combined in one 24-hour period

The median LOS was 130.0 min (interquartile range across EDs: 79.0–194.0). Figure 1 shows that there was a peak in LOS at the end of the night and a slightly smaller peak at the end of the afternoon. The majority of patients presented in the afternoon between 12 PM and 5 PM. The median LOS was longest for patients presenting with a non-trauma related problem (151 min), patients presenting during week-days (142 min), patients referred by medical professionals (148 min) and patients who were admitted to hospital (169 min). For patients referred by the radiology department the median LOS was shortest with 71.5 min (Table 3).

**Table 3:** Length of stay (LOS) stratified by patient characteristics and care pathways

*N* = 7000		Median LOS (in minutes)	Interquartile range
Overall median LOS		130.0	79.0–194.0
Gender	Male	134.5	84.0–198.0
	Female	127.0	74.0–191.0
Trauma*	Yes	91.0	52.0–141.0
	No	151.0	99.0–215.0
ECAP	Yes	136.0	82.0–200.0
	No	125.5	76.0–190.0
Time of presentation*	Weekdays^a^	142.0	86.0–210.0
	Evenings	123.0	77.0–176.0
	Nights	116.0	69.5–172.0
	Weekend days	126.0	72.0–199.0
Origin* *N* = 6908^§^	Self-referred patients^a^	99.0	54.0–154.0
	Referred by medical professionals^b^	148.0	97.0–212.0
	Referred by the radiology department	71.5	44.0–125.0
Follow-up*	Hospital Admission^a^	169.0	120.0–238.0
	Out patient Clinic	108.0	66.0–164.0
	Primary Care Physician	122.0	79.0–179.5
	None	93.0	53.3–145.0

*P < 0.05 in the regression analysis, ^a^compared parameter in the random coefficient regression analysis ^§^There were 92 missing data on patients origin

The random coefficient regression analysis showed that patients presenting with a trauma related problem had a 51 min shorter LOS (95 % CI, 46.6–55.6 min) compared to patients with a non-trauma related problem.

LOS increased with age (p 0.00). There was no association with LOS and sex. Compared to presentations during weekdays, LOS was significantly shorter for presentations in the evening (21.1 min, 95 % CI 15.8–26.4), night (25,2 min, 95 % CI 18.1–32,2) and on weekend days (15.8 min, 95 % CI 9.1–22.5) Compared to self-referred patients, LOS was significantly shorter for patients referred via the radiology department (14.9 min, 95 % CI 2.2–22.5) and significantly longer for patients referred by medical professionals (57.3 min, 95 % CI 52.0–62.5). Compared to patients requiring a hospital admission, there was a significantly shorter LOS for patients followed-up in an out-patient clinic (63.8 min, 95 % CI 58.6–69.1), followed-up by their PCP (72.2 min, 95 % CI 63.7–80.7) or who did not need any follow-up (71.3 min, 95 % CI 65.2–77.7).

Regarding the moment of presentation, the LOS was longest on weekdays. After correcting for age, sex and trauma, the mean LOS was 13.1 min shorter in the evening (95 % CI, 8.2–18.1 min, P 0.00), 20.4 min shorter at night (95 % CI, 13.9–27.0 min, P 0.00) and 11.0 min shorter on weekend days (95 % CI, 4.7–17.2 min, P 0.001). Compared to self-referred patients, only the LOS for patients referred by medical professionals remained significantly longer, with 32,9 min (95 % CI 27.7–38.2 min, P 0.00). For follow-up, LOS was longest for patients requiring a hospital admission. LOS remained significantly shorter with 41.2 min for patients followed up at outpatient clinics (95 % CI 35.4–46.2 min, P 0.000), 49.9 min shorter for patients followed up with their PCP (95 % CI 40.3–57.1 min, P0.000) and 44.6 min shorter for patients who did not need any follow-up (95 % CI 43.2–55.1 min, P0.000) (Table 4). There was no significant difference in LOS between ECAP and non-ECAP hospitals after-hours.

**Table 4:** Factors associated with LOS^a^

		Before including fixed effects^b^				After including fixed effects^b^			
		Difference in LOS^c^	95 % Confidence Interval		*P* value	Difference in LOS^c^	95 % Confidence interval		*P* value
			Lower bound	Upper bound			Lowerbound	Upper bound	
Time of presentation	Weekday^c^	Reference				Reference			
	Evening	−21.1	−26.4	−15.8	0.000	−13.1	18.1	−8.2	0.000
	Night	−25.2	−32.2	−18.1	0.000	−20.4	−27.0	−13.9	0.000
	Weekend day	−15.8	−22.5	−9.1	0.000	−11.0	−17.2	−4.7	0.001
Origin	Self referred^c^	Reference				Reference			
	Referred by medical professionals	57.3	52.0	62.5	0.000	32.9	27.7	38.2	0.000
	Referred via radiology	−14.9	−27.6	−2.2	0.022	−6.5	−18.7	5.6	0.293
Follow up	Hospital Admission^c^	Reference				Reference			
	Out patient Clinic	−63.8	−69.1	−58.6	0.000	−41.2	−46.6	−35.8	0.000
	PCP Follow-up	−72.2	−80.7	−63.7	0.000	−49.9	−58.3	−41.5	0.000
	No Follow-up	−71.3	−77.7	−65.2	0.000	−44.6	−51.0	−38.3	0.000

Legend. ^a^Results from random coefficient regression modeling, all analysis included hospitals as random effect, ^b^fixed effects are age, sex and trauma, ^c^difference in LOS compared to the reference category

### Characteristics of factors associated with different LOS

Of 6908 patients (92 had missing data), 21.2 % was self-referred (median LOS 99.0 min), 59.9 % was referred by medical professionals (median LOS 148.0 min) and 3.3 % presented via the radiology department (median LOS 71.5 min). The mean age for self-referrals was 37.5 years and for patients referred by medical professionals 50.9 years old. Of the self-referrals, 51.6 % presented with trauma related symptoms and the majority (56.1 %) was aged between 16 and 50 years old. A total of 11.4 % of the self-referrals required hospital admission. Patients referred by medical professionals presented with mostly non-trauma related symptoms (73.1 %) and the majority was aged between 51 and 85 years old. With 48,9 %, a much higher percentage of these patients required admission. Only 3.5 % of patients referred by the radiology department required a hospital admission and 75.6 % was followed up at the outpatient clinic (Table 5).

**Table 5:** Characteristics of patient groups

		Self-referrals (*N* = 1877)	Referrals	
			Medical professionals (*N* = 4138)	Radiology department (*N* = 230)
Total (%)		21.2	59.9	3.3
Male (%)		57.5	49.6	50.0
Age (%)	0–5 years	6.2	8.0	2.2
	6–15	8.6	4.6	18.3
	16–30	29.6	13.2	16.1
	31–50	26.5	18.5	19.6
	51–65	16.6	20.5	23.5
	66–85	10.8	28.4	15.2
	>85	1.7	6.8	5.2
Presentation (%)	Weekday^b^	38.0	40.9	92.6
	Evening^c^	31.5	30.9	4.8
	Night ^d^	14.2	14.1	1.3
	Weekend day^b^	16.2	14.1	1.3
Follow-up (%)^a^	Hospital Admission	11.4	48.9	3.5
	Out patient Clinic	29.4	24.4	75.6
	PCP Follow-up	18.6	6.2	3.0
	No Follow-up	31.6	17.5	12.2
Trauma (%)		51.6	23.4	89.6
Median LOS (minutes)		99.0	148.0	71.5
*N* = 4070^f^		ECAP (*N* = 1785)	Non-ECAP (*N* = 2285)	
Male (%)		51.9	53.1	
Age (%)	0–5 years	6.8	8.9	
	6–15 years	4.4	7.6	
	16–30 years	19.4	22.3	
	31–50 years	21.2	21.8	
	51–65 years	19.3	17.5	
	66–85 years	23.7	17.9	
	>85 years	5.3	4.0	
Time of presentation	Evenings^c^	49.9	53.0	
	Nights^d^	23.1	22.1	
	Weekend days^b^	27.0	24.9	
Origin (%)^a^	Self-referred patients	15.6	38.7	
	Referred by medical professionals^e^	74.1	49.1	
	Referred by the radiology department	0.5	0.4	
Follow-up (%)^a^	Hospital Admission	42.7	31.7	
	Out patient Clinic	27.7	26.7	
	Primary Care Physician	5.0	13.3	
	None	21.0	23.2	
Median LOS (minutes)		129.0	118.0	
Trauma (%)		28.8	35.8	

^a^Does not add up to 100 % due to other options not shown in the table ^b^8 am-5 pm, ^c^5 pm-12 am, ^d^12 am-8 am, ^e^Includes ambulance and PCP referred patients ^f^ N during opening hours ECAP

During ECAP opening hours, the median LOS in ECAP EDs was 129 min compared to 118 min in non-ECAP EDs. In ECAP EDs the average age was 47.6 years old with the majority between 66 and 85 years. In non-ECAP EDs the average age was 42.1 years old with the majority aged between 16 and 30 years old. In ECAP EDs, 15.6 % were still registered as self-referred patients compared to 38.7 % in non-ECAP EDs. These patients presented to the ECAP unannounced and were registered for the ED after triage. For patients referred by medical professionals this was 74.1 % compared to 49.1 %. In ECAP hospitals 42.7 % of patients required an admission compared to 31.7 % in non-ECAP hospitals (Table 5).

## Limitations

Though the data was extracted from digital hospital systems, they included self-reported data, which could have caused inaccuracies. Furthermore this study did not involve academic centers. The data does however represent the overall Dutch healthcare system with EDs from seven different regions. When combing patients referred by PCPs and patients arriving by ambulance in the same group, there is a small percentage of self-referrals in that group. Ambulances, however, have the option not to transport the patient to the ED and instead have patients use their own mode of transport or contact the patients PCP and hand over treatment. This makes the percentage of low-acuity patients in the combined group small and therefore it seems plausible to combine them. Since there were three different triage systems in use, we could unfortunately not compare patients triage in association with LOS. The same applies to differences in trauma scores. To the best of our knowledge this is the first research in Dutch EDs exploring the influence of a strong primary care system on LOS.

## Discussion

This study assessed the relationship between length of stay and patients’ care pathways in hospital EDs in the Netherlands. It showed an overall median LOS of 130 min. Factors associated with a longer LOS were older age, presentation during weekdays, referral by medical professionals and hospital admission. A factor associated with a shorter LOS was linked to patients who underwent ancillary tests prior to ED presentation. A median LOS of 130 min is relatively short compared to internationally published estimates of LOS, which had median values from 176 to 480 min [19–21]. Our data showed a shorter median LOS for both admitted patients and discharged patients compared to the United States for similar sized EDs, hospitals with the same number of in-hospital beds and EDs with more than 20 % trauma related problems [22, 23]. Factors related to a longer LOS probably represent similar patients, for instance patients being admitted to the hospital are often older and referred by a medical professional. This group may benefit from organizational improvements at the ED such as fast tracks [24, 25]. To guarantee patient safety in such fast tracks more insight into risk factors is needed, which indicate a need for more extensive diagnostic procedures (i.e. abdominal pain in an elderly patient).

Our study showed a significant shorter LOS for patients referred via the radiology department compared to other origin. In the Netherlands, it is a common procedure during office hours and at several ECAPs to refer the patient directly to the radiology department for ancillary testing. If an abnormality is found, the patient will then be referred to the ED. When no abnormalities are found, the patient will not present at the ED but will be referred back to the PCP [14]. Implementing this possibility for all PCPs and ECAPs could further reduce the overall median LOS for ED attendances that require an ED visit since tests have already been performed. Furthermore, it will decrease the number of unnecessary referred patients, should a test be negative. If PCPs also have the possibility to perform diagnostic laboratory tests after-hours as they do during office hours, this would even further reduce the number of referred patients.

This study showed an overall admission rate of 36.3 % of all ED attendances, which is higher in percentage compared to the United States (US) and the United Kingdom (UK) [26, 27]. With only 11.4 % of the already small group of selfreferrals (21.2 %) being admitted compared to 48.9 % of patients being referred by their PCP, it suggests that PCPs function well as gatekeepers to the ED.

There are several factors identified as causes for crowding and all of them could explain the relatively short LOS in Dutch EDs. One of the factors is the non-urgent ED patient. In the Netherlands approximately 1.9–2.2 million patients visit the ED yearly, around 124–135 visits per 1000 inhabitants [28]. This is low compared to other countries like the United States, 405–428 per 1000 inhabitants, Canada, 470 per 1000 inhabitants and the UK where 396 per 1000 inhabitants visit the ED yearly [29–32]. Good access to quality primary care seems the key reason for the large difference in ED visits.

In the Netherlands, primary healthcare is well-developed and accessible for patients 24 h a day. During office-hours patients can see their own PCP, usually on the same day. After-hours, PCPs provide emergency services through large scale PCP-cooperatives [12]. In the United States, where the median LOS is longer compared to the Netherlands, access to primary care is not readily available for everyone. In fact, the percentage of PCPs providing after-hours care is only 29 % compared to almost a 100 % in the Netherlands [10]. As a result, United States EDs may increasingly serve as a safety net with increasing numbers of patient visits. The average yearly number of 26.666 ED patient visits in the United states (total annual yearly ED visits divided by total number of national EDs) is high compared to the Netherlands, where an average of 22.448 patients visit each ED per year. These differences in healthcare systems make comparison of LOS difficult. It does however show that primary care, resulting in a low percentage of self-referrals, leads to a shorter LOS.

Another factor associated with crowding is the number of hospital beds. The Netherlands has 4.7 beds per 1000 inhabitants compared to 3.0 per 1000 inhabitants in the United States and the United Kingdom and 3.2 beds per 1000 inhabitants in Canada [33]. It seems that the overall healthcare system in the Netherlands plays a large role in the shorter LOS.

We did not find a significant difference in LOS after-hours between hospitals with and without an ECAP. Hospitals with an ECAP see more referred patients and more patients requiring an admission, both factors associated with a longer LOS [14]. When analyzing 1000 consecutive patients in non-ECAP EDs with a higher percentage of self-referrals, and comparing them with 1000 consecutive patients in ECAP EDs, which are mostly, referred patients, a similar LOS might assume that it is not the illness severity of the patient that is predictive for the LOS, but rather the ED procedure. The intention was also to compare LOS of patients with a different acuity, but due to three different triage systems this was not possible. Because diagnostic tests are ordered for 65 % of the non-urgent patients and 95 % of the urgent patients in Dutch EDs, It seems plausible that there is no difference in performed diagnostic tests between the self-referral and the referred patient [34]. Although crowding is mentioned as a problem by ED managers in a web-based survey, factors associated with LOS in EDs in the Netherlands were never studied.

## Conclusion

This study showed that LOS in EDs in the Netherlands is relatively short compared to other countries, which is probably due to its well-developed primary care system. LOS was longer for older patients, patients referred by medical professionals and patients who required a hospital admission. With the number of ECAPs increasing, LOS can perhaps decrease, by strengthening primary healthcare even more, through implementing PCP access to ancillary services like radiology and laboratory tests and by collaboration guidelines between PCP and ED care. Gaining insight in presenting complaints and performed diagnostic tests seems crucial to develop these guidelines and implement fast tracks to reduce LOS.

## Ethics approval and consent to participate

The nationally recognized medical ethical committee of the Catharina hospital granted institutional review board exemption.

## Consent for publication

Not applicable.

## Availability of data and materials

Data will not be shared at this time, since further research is still being done. After completion, it will be made available.

## Abbreviations

EDs: emergency departments
LOS: length of stay
PCP: Primary-Care Physician
ECAP: emergency care access point
PCI: primary cardiac interventions
MTS: Manchester Triage System
ESI: emergency severity index
NTS: Netherlands Triage Standard
US: United States
UK: United Kingdom
